# Gene Signature of Regulatory T Cells Isolated from Children with Selective IgA Deficiency and Common Variable Immunodeficiency

**DOI:** 10.3390/cells13050417

**Published:** 2024-02-27

**Authors:** Magdalena Rutkowska-Zapała, Agnieszka Grabowska-Gurgul, Marzena Lenart, Anna Szaflarska, Anna Kluczewska, Monika Mach-Tomalska, Monika Baj-Krzyworzeka, Maciej Siedlar

**Affiliations:** 1 Department of Clinical Immunology, Institute of Paediatrics, Jagiellonian University Medical College, Wielicka 265, 30-663 Krakow, Poland; 2 Department of Medical Genetics, Institute of Paediatrics, Jagiellonian University Medical College, Wielicka 265, 30-663 Krakow, Poland; aga.grabowska@uj.edu.pl; 3 Department of Clinical Immunology, University Children’s Hospital, Wielicka 265, 30-663 Krakow, Poland; mmach@usdk.pl

**Keywords:** common variable immunodeficiency, selective IgA deficiency, regulatory T cells, microarray analysis

## Abstract

Selective IgA deficiency (SIgAD) is the most common form and common variable immunodeficiency (CVID) is the most symptomatic form of predominant antibody deficiency. Despite differences in the clinical picture, a similar genetic background is suggested. A common feature of both disorders is the occurrence of autoimmune conditions. Regulatory T cells (T_regs_) are the major immune cell type that maintains autoimmune tolerance. As the different types of abnormalities of T_reg_ cells have been associated with autoimmune disorders in primary immunodeficiency (PID) patients, in our study we aimed to analyze the gene expression profiles of T_reg_ cells in CVID and SIgAD patients compared to age-matched healthy controls. The transcriptome-wide gene profiling was performed by microarray technology. As a result, we analyzed and visualized gene expression patterns of isolated population of T_reg_ cells. We showed the differences at the gene level between patients with and without autoimmunizations. Our findings suggest that the gene signatures of T_reg_ cells isolated from SIgAD and CVID patients differ from age-matched healthy controls and from each other, presenting transcriptional profiles enriched in innate immune or Th response, respectively. The occurrence of autoimmunity in both types of PID is associated with down-regulation of class I IFNs signaling pathways. In summary, our findings improve our understanding of T_reg_ dysfunctions in patients with common PIDs and associated autoimmunity.

## 1. Introduction

Selective IgA deficiency (SIgAD) and common variable immunodeficiency (CVID) belong to the group of inborn errors of immunity, being the most common and the most symptomatic forms of predominant antibody deficiency, respectively. The occurrence of both diseases within one family and observed progression of SIgAD to CVID suggests a similar genetic background [1]. SIgAD occurs with the highest prevalence, ranging from 1/142 to 1/965 in the Caucasian population, and in the lowest frequency of 1/18,550 among the Japanese population [2]. SIgAD is defined as serum IgA concentration lower than 0.07 g/L and normal IgM and IgG levels in children aged 4 years or older, in which other causes of immunodeficiency were excluded [3]. Patients with SIgAD demonstrate heterogeneous phenotypes. Most individuals (approx. 85–90%) are clinically asymptomatic at the time of diagnosis; however, recurrent sinopulmonary and gastrointestinal infections were reported in both children and adults [4,5,6,7]. This disease does not follow a simple Mendelian inheritance pattern; however, it exhibits familial clustering. The prevalence of CVID ranges from 1/10,000 to 1/100,000 of the population, affecting approximately 1/25,000 Caucasians, and appears to be the most frequent form of PID in adults [8]. CVID patients have a marked reduction in serum concentration of both IgG (<3 g/L) and IgA (<0.05 g/L), while IgM is reduced (<0.3 g/L) in about half of the patients. Moreover, a reduced or absent antibody response to vaccination was observed [9]. Clinical manifestations of CVID primarily include recurrent sinopulmonary infections [10]. Patients may also present with an increased predisposition to the development of cancer, autoimmunity, or inflammatory disorders [10]. A number of monogenic forms of CVID were described; nonetheless, they explained only a minority of CVID cases [11].

A common feature of both SIgAD and CVID, which might also be their first clinical manifestation, is the occurrence of autoimmune conditions that affect approximately 25.5% to 31.7% of SIgAD patients and about 30% of CVID patients [2,12]. However, the autoimmune phenotype in these patients may be atypical, causing a delay in the final diagnosis. Therefore, there is a need to identify biomarkers of autoimmune complications in PID patients. Regulatory T cells (T_regs_) may become a promising parameter for this type of analysis. T_regs_ play a pivotal role in retaining immune tolerance and homeostasis by suppressing effector T cells’ and antigen-presenting cells’ functions [13]. Disorders in T_reg_ development and function are associated with a variety of autoimmune phenomena. T_reg_ abnormalities are caused by defects in key T_reg_ genes, such as FOXP3 and IL2RA (IPEX and IPEX-like syndrome, respectively), CTLA4, STAT5B, and IL2RB [14]. In addition, many primary immunodeficiencies are associated with impaired T_reg_ number or function, like Omenn syndrome, calcium channel defects (ORAI1 and STIM1 deficiency), and DOCK8, WASP, and RLTPR deficiencies [15,16]. In CVID, the role of T_regs_ in disease development and progression was considered. Several studies showed a lower frequency of T_reg_ cells in patients with CVID; however, contradictory results were also published [17,18,19,20,21,22]. The reason for these inconsistent results might be associated with autoimmune complications that occur in some, but not all, CVID patients [23,24,25,26,27]. Thus, the aim of our study was to analyze the transcriptome signature of T_reg_ cells in CVID and SIgAD patients compared with healthy controls as well as between CVID and SIgAD subgroups, comparing patients with or without autoimmune disorders/presence of autoantibodies. 

Our results provide noteworthy data to better understand T_reg_ dysfunction observed in patients with common primary humoral immunodeficiencies and improve our knowledge of the role of T_reg_-associated genes in the etiopathogenesis of autoimmune diseases in CVID and SIgAD.

## 2. Materials and Methods

### 2.1. Patients

**Figure 1:**
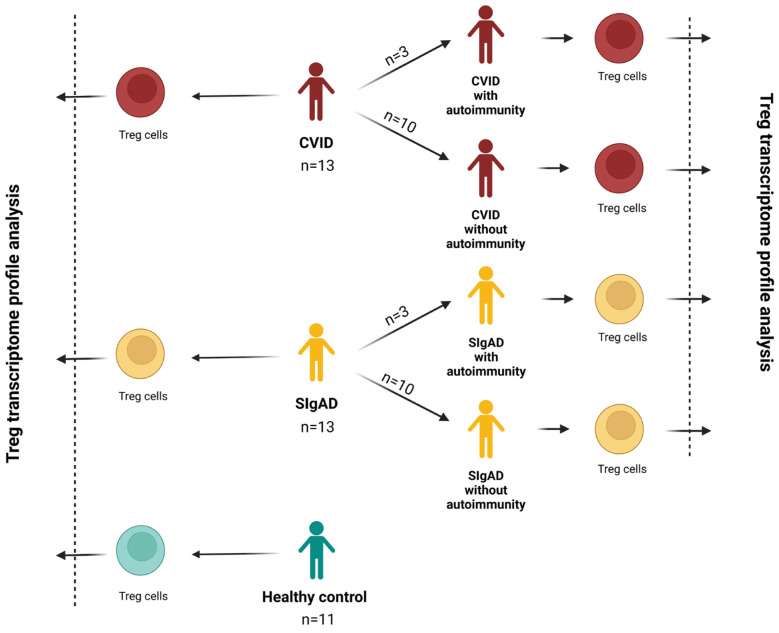
The scheme of the study.

We studied a cohort of 26 PID patients and 11 healthy control subjects. The diagnosis of CVID and SIgAD was based on the European Society for Immunodeficiencies (ESID) criteria [28]. We enrolled 13 patients with CVID receiving regular immunoglobulin replacement therapy. Among them, three patients had accompanying diseases such as thrombocytopenias and ulcerative colitis. The SIgAD group consisted of 13 patients, including 3 patients with autoimmunization diseases such as celiac disease, juvenile arthritis, or Sjögren disease. The characteristic details of the studied groups are presented in Table 1, while the scheme of the study design is presented in Figure 1. All patients were treated in the outpatient units of the Department of Clinical Immunology of the University Children’s Hospital in Krakow. The study was approved by the Bioethical Committee of Jagiellonian University (122.6120.2.2015 of 29 January 2015). Written informed consent was obtained from all the study participants.

### 2.2. Regulatory T Cells Number Evaluation

Whole peripheral blood samples from PID patients and healthy controls were drawn into tubes containing EDTA (Vacutainer System; Becton Dickinson, Franklin Lakes, NJ, USA). For T cell number evaluation, whole blood samples were incubated with anti-CD3-FITC and anti-CD4-PE (BD Biosciences, San Jose, CA, USA) monoclonal antibodies (mAb) in TruCount tubes (BD Biosciences), lysed, and analyzed on a flow cytometer (FACSCanto; Becton Dickinson Immunocytometry Systems, Palo Alto, CA, USA). The absolute numbers of CD3^+^CD4^+^ T cells were calculated on the basis of bead and lymphocyte counts. For absolute T_reg_ number evaluation, peripheral blood mononuclear cells (PBMC) from the same person were isolated by the standard Ficoll-Paque (Pharmacia Biotech) density gradient centrifugation. Then, PBMCs were stained using the Human Regulatory T cell Staining Kit (eBiosciences, San Diego, CA, USA). The T_reg_ absolute numbers calculation was based on the percentage of T_regs_ among CD4^+^ lymphocytes and the absolute number of CD3^+^CD4^+^ T cells. The gating strategy for T_reg_ cell FACS analysis is presented in Supplementary Figure S1. T_reg_ lymphocytes are considered to express CD4, CD25, and Foxp3 antigens simultaneously.

### 2.3. Isolation of Regulatory T Cells

T_reg_ cells were isolated by magnetic sorting from PBMCs using a two-step procedure using a Regulatory T Cell Isolation Kit II (Miltenyi Biotech, Tokyo, Japan) according to the manufacturer’s protocol. Briefly, cells were incubated with a cocktail of biotinylated antibodies and Anti-Biotin MicroBeads for the depletion of non-CD4^+^ and CD127^high^ cells. Then, the flow-through fraction of pre-enriched CD4^+^CD127^dim/−^ T cells was incubated with CD25 MicroBeads for subsequent positive selection of CD4^+^CD25^+^CD127^dim/−^ T_reg_ cells. LD and MS Columns (Miltenyi Biotech) were used during the first (depletion) and second (positive selection) magnetic separations, respectively. Then, cells were washed in MACS buffer, centrifuged for 10 min at 350× *g*, and frozen at −80 °C until RNA isolation.

### 2.4. Gene Expression Analysis

The analysis of transcriptome-wide gene expression profiles was performed for T_reg_ cell populations isolated from PID patients and healthy controls using microarray technology and Clariom D Assays (Affymetrix, Santa Clara, CA, USA). In brief, total RNA was extracted from isolated T_reg_ cell populations using an RNeasy Micro Kit (Qiagen, Hilden, Germany) according to the manufacturer’s protocol. The RNA concentration and quality was analyzed on a NanoDrop 1000 Spectrophotometer (ThermoScientific, Waltham, MA, USA). The input material quantity was 50 ng of total RNA. Array hybridization was processed with a GeneChip WT PLUS Reagent Kit and a GeneChip 3000 instrument system (Affymetrix). The Transcriptome Analysis Console (TAC) Software (Affymetrix) was used to analyze raw data for quality and gene expression patterns. The gene advanced Robust Multiarray Analysis method with Signal Space Transformation (SST-RMA) summarization was performed by TAC (version 4.0.2.15 for Windows, Waltham, MA, USA, www.thermofisher.com, (accessed on 22 December 2023)). The quality of the experiment was determined on the basis of the values of Pos vs. Neg AUC. The following filter criteria were applied: a fold change > 2 or <−2 and a *p*-value < 0.05. GO enrichment and KEGG (Kyoto Encyclopedia of Genes and Genomes) pathway analysis of targeted genes were performed using the Database for Annotation, Visualization and Integrated Discovery (DAVID) online tools [29]. Microarray data will be submitted to the GEO database.

### 2.5. Quantitative Real-Time PCR (RT-qPCR) Analysis

Microarray results were validated by the RT-qPCR method and the TaqMan method. Briefly, reverse transcription was performed using SuperScriptIII First-Strand Synthesis SuperMix (Invitrogen, ThermoFisher Scientific, Waltham, MA, USA). PCR reactions were performed in duplicates using TaqMan Gene Expression Master Mix (ThermoFisher Scientific, MA, USA) and appropriate assays containing the following primers and probes (ThermoFisher Scientific, USA): Eukaryotic 18S rRNA (assay ID: Hs03003631_g1), FOXP3 (assay ID: Hs01085834_m1), LEF1 (assay ID: Hs01547250_m1), and MAPK3 (assay ID: Hs00385075_m1). The RNA samples that underwent microarray analysis were used for qPCR. RT-qPCR was performed on a QuantStudio 7 System (Applied Biosystems, Waltham, MA, USA). The relative amounts of mRNAs were calculated using the 2^−ΔΔCT^ method and 18S was used as a control for each PCR run.

### 2.6. Statistical Analysis

Statistical analysis was performed using GraphPad Prism version 8 (GraphPad Software Inc., San Diego, CA, USA). The differences between two groups were analyzed using a Student’s *t*-test or Mann–Whitney test where applicable. The normal distribution of values was verified using a Shapiro–Wilk test. For multiple comparisons, a non-parametric Kruskal–Wallis test with Dunn’s post hoc test was applied. For parametric results, mean ± standard error of mean (SEM) was shown, while for nonparametric results, median ± interquartile range (IQR: Q1-25%, Q3-75%) was shown. The *p* values < 0.05 were considered significant.

## 3. Results

### 3.1. Regulatory T Cells Number

**Figure 2:**
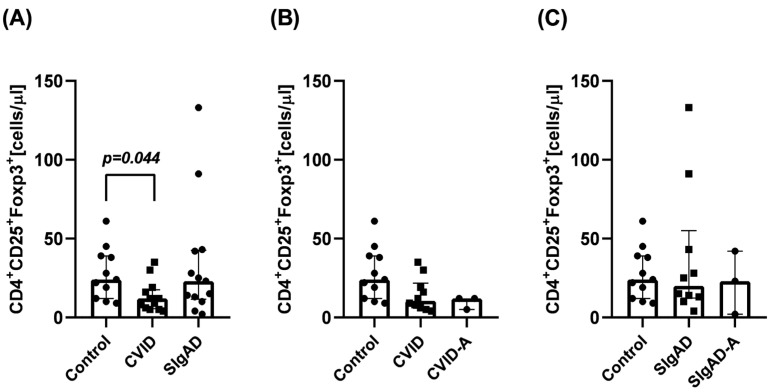
T_reg_ numbers in patients with CVID or SIgAD and healthy subjects. T_reg_ numbers were analyzed in the whole CVID or SIgAD and control groups (**A**), and the the patient groups were divided into subgroups, with (**B**) and without (**C**) autoimmune diseases. The differences between studied groups were analyzed using a Kruskal–Wallis test and median with interquartile range is shown.

Firstly, we analyzed the absolute numbers of T_reg_ cells in children with SIgAD or CVID and healthy controls. T_reg_ levels were significantly (*p* = 0.044) reduced only in children with CVID when compared with healthy controls (Figure 2A). The median T_reg_ number in the control group was 24 (IQR: 12; 39), in the CVID group was 12 (IQR: 6; 18), and in SIgAD the median T_reg_ number was 23 (IQR: 12; 43). Next, T_reg_ numbers were analyzed in both PID groups, which were divided into two subgroups: with (CVID-A, SIgAD-A) and without (CVID, SIgAD) the accompanying autoimmune diseases (Figure 2B,C). Within the CVID (Figure 2B) and SIgAD (Figure 2C) subgroups, the absolute numbers of circulating CD4^+^CD25^+^Foxp3^+^ cells were comparable in all studied groups. The median T_reg_ number in the CVID subgroup was 11 (IQR: 6; 12), in the CVID-A subgroup was 12 (IQR: 5; 12), in the SIgAD subgroup was 20 (IQR: 12; 55), while in the SIgAD subgroup it was 23 (IQR: 2; 42). 

### 3.2. Gene Expression Analysis in T_reg_ Cells

**Figure 3:**
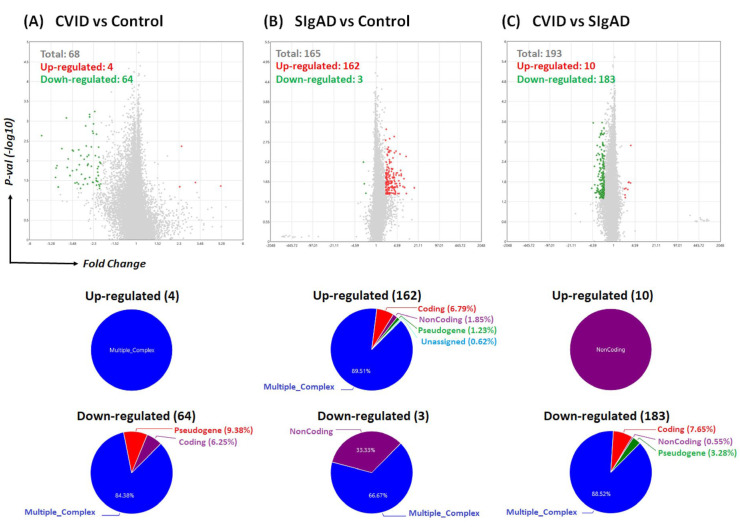
Results of microarray analysis of T_reg_ cell gene expression profiles in comparisons: CVID vs. control (**A**), SIgAD vs. control (**B**), and CVID vs. SIgAD (**C**). Volcano plots show differentially expressed transcripts (*p*-values < 0.05)—red spots represent up-regulated, while the green ones represent the down-regulated genes. In grey—non-significantly differentially expressed genes are represented. The pie graphs below each volcano plot show up-regulated (**top**) and down-regulated (**bottom**) genes affiliated with particular groups of transcripts.

Our strategy for T_reg_ expression profile analysis was bidirectional. Firstly, we performed analysis for all studied groups, i.e., SIgAD, CVID, and healthy control, and the comparisons were performed in pairs. A graphical representation of differentially expressed genes (DEGs) in all mentioned comparisons, including volcano plots and pie graphs, are presented in Figure 3. These comparisons show that T_reg_ cells isolated from the CVID patients and healthy subjects groups differed the least. Among 68 DEGs, 4 were up- and 64 were down-regulated in CVID when compared to the control group. Most of the detected DEGs—all the up-regulated and the majority of the down-regulated (84.38%)—belonged to multiple complex groups (Figure 3A). T_regs_ isolated from SIgAD patients seemed to differ slightly more than those from CVID patients when compared with the control group. In that case, among 165 DEGs, 162 were up- and 3 were down-regulated in the SIgAD group when compared to the control group (Figure 3B). Among up-regulated genes, the majority belonged to multiple complex (89.51%) or coding groups (6.79%). The highest number of DEGs was detected when T_reg_ cells from both groups of PID patients were compared. Here, among 193 DEGs, 10 were up- while 183 were down-regulated in CVID patients when compared to SIgAD patients (Figure 3C). All from up-regulated genes were noncoding, while most of the down-regulated genes belonged to multiple complex groups (88.52%).

**Figure 4:**
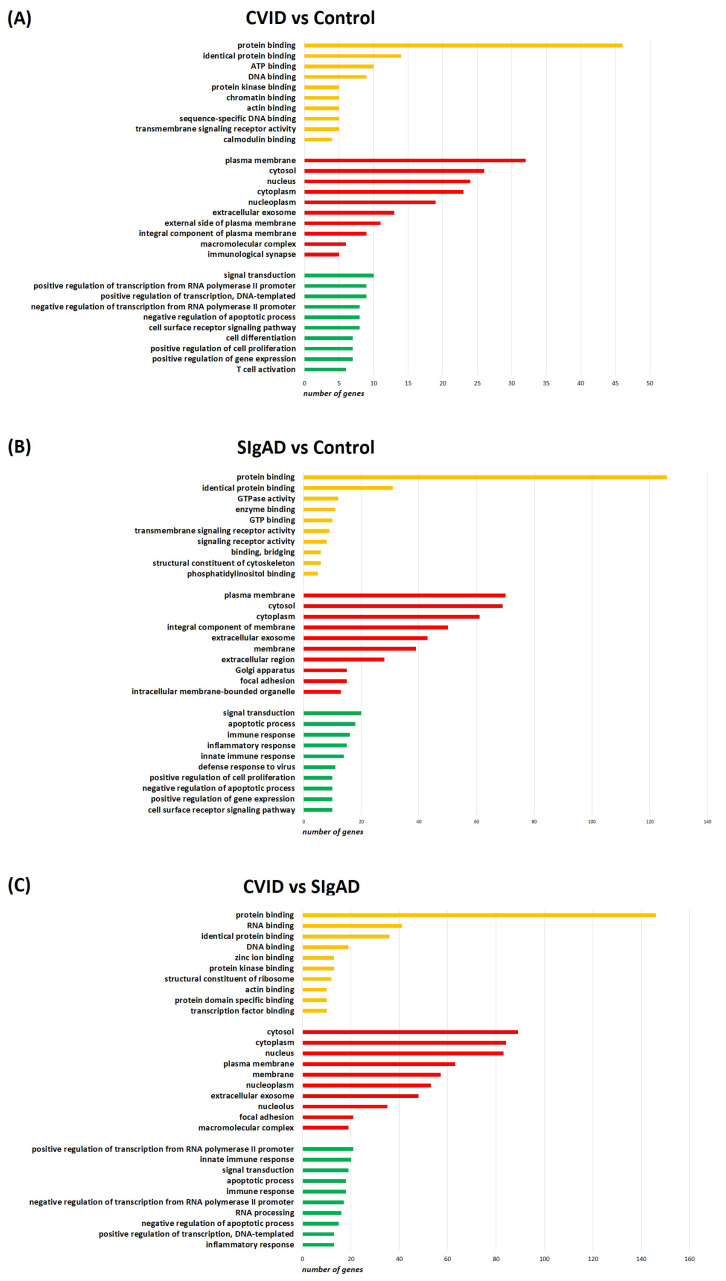
Top 10 Gene Ontology (GO) annotation categories in performed comparisons on CVID vs. control (**A**), SIgAD vs. control (**B**) and CVID vs. SIgAD (**C**), presented as bar charts, including molecular function (in yellow), cellular component (in red), and biological process (in green).

To investigate the biological role of the DEGs’ detected comparisons, enrichment analysis was performed using the DAVID database. The bar chart depicts the top 10 (GO) annotation categories, such as biological and molecular functions and cellular components, and is presented in Figure 4. Regarding components common for all comparisons, we observed that molecular function had the majority of genes distributed across functions such as protein binding and identical protein binding. In terms of cellular components, DEGs were mainly associated with the plasma membrane, cytosol, cytoplasm, and extracellular exosome. Meanwhile, the key genes are related to biological processes associated with signal transduction and negative regulation of apoptosis. Regarding biological processes, we also observed some differences in both immunodeficiencies when compared with the control group. In patients with CVID, DEGs appeared to be associated with T cell activation, which was not observed in the SIgAD group. Conversely, in patients with SIgAD, DEGs appeared to be associated with the innate immune response, which was not observed in CVID.

**Figure 5:**
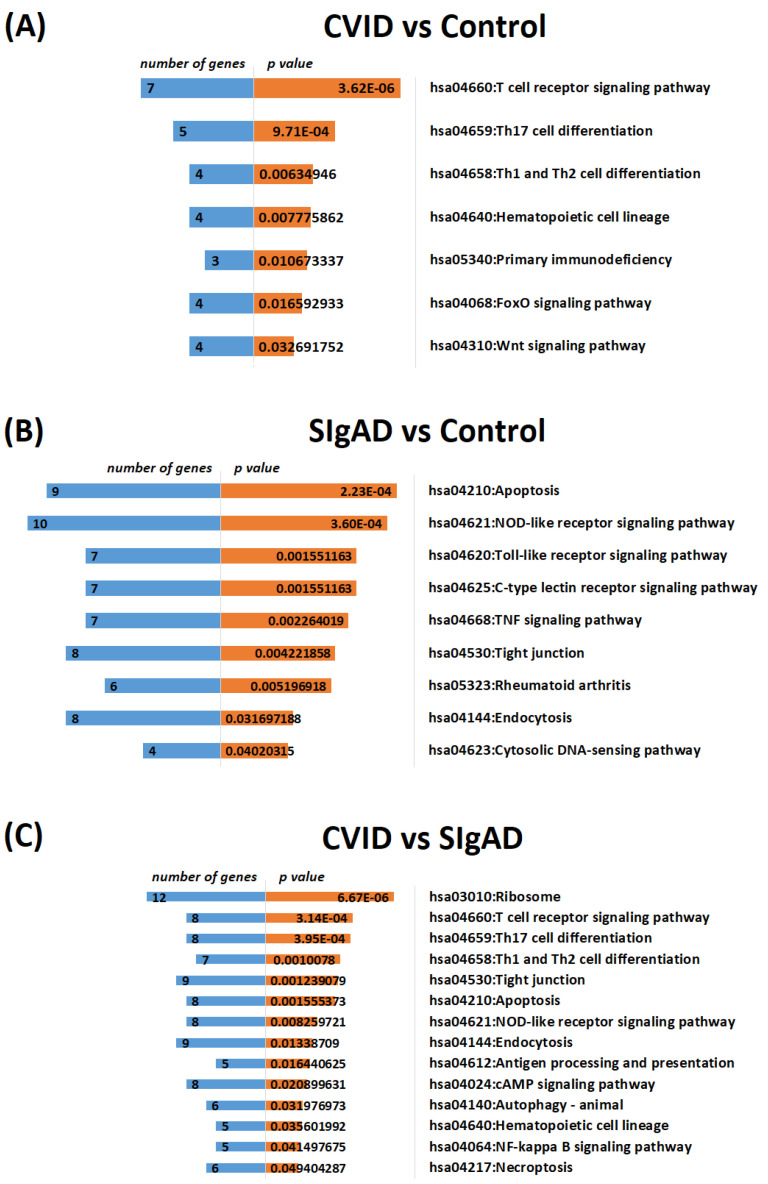
Top enriched KEGG pathways of DEGs, demonstrated by number of genes (in blue) and *p*-value (in orange). Analysis was performed in CVID vs. control (**A**), SIgAD vs. control (**B**) and CVID vs. SIgAD (**C**) comparisons using the default settings (count = 2, EASE = 0.1).

To explore the signaling pathways of DEGs, KEGG pathway analysis was performed via the DAVID database. The graphical representation of the pathway enrichment analysis is shown in Figure 5. CVID patients’ analyses, compared to healthy controls, as well as to the SIgAD group, showed that DEGs were primarily enriched in the T cell receptor signaling pathway and associated with Th17 cell differentiation. However, DEGs detected in the comparison of SIgAD patients with healthy subjects seemed to be enriched in the phagosome, apoptosis, the NOD-like receptor signaling pathway, and rheumatoid arthritis.

**Figure 6:**
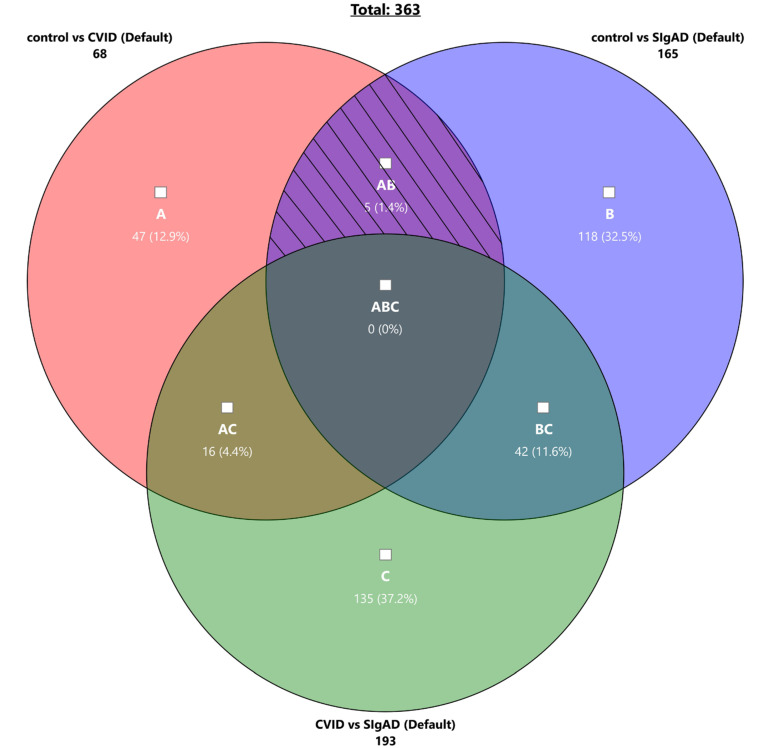
Venn diagram showing the overlap of the analyzed pairs of analyzed groups. Each circle represents genes that are differentially expressed in analyzed comparisons, i.e., CVID vs. Control (**A**), SIgAD vs. Control (**B**), and CVID vs. SIgAD (**C**). Areas where the circles overlap indicate characteristics shared between two or more data sets. The hatched field indicates genes that are differentially expressed both in CVID and SIgAD patients when compared to healthy controls.

The common DEGs of all analyzed comparisons were evaluated by a Venn diagram (Figure 6). The Venn diagram revealed no shared differential genes between all mentioned comparisons. Nevertheless, five genes (*CAMK4*, *IL6ST*, *OGFRL1*, *ATP6V1B2*, and *TNFAIP2*) seem to be differentially expressed both in CVID and SIgAD patients when compared to healthy controls. Moreover, the gene expression regulation pattern is similar in PID patients when compared with controls, as *CAMK4* and *IL6ST* were down-regulated, while *OGFRL1*, *ATP6V1B2*, and *TBNAIP2* were up-regulated in CVID and SIgAD patients. Short characteristics of these genes are presented in Table 2.

**Figure 7:**
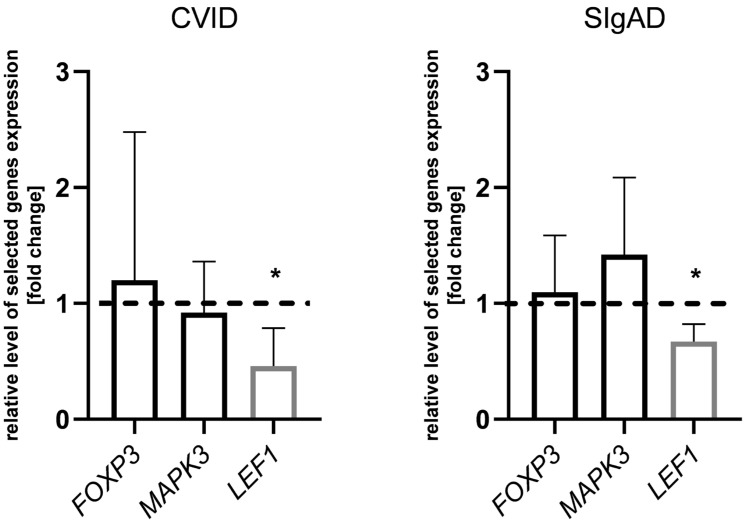
Validation of microarray results. Relative expression of randomly selected genes (*FOXP3*, *MAPK3*, and *LEF1*) validated by qPCR, presented as fold change of each DEG’s relative expression, normalized to S18 expression and the healthy control group (2^−ΔΔCT^). The results were obtained from individual real-time PCR reactions performed with CVID and SIgAD cells. Dashed line was set on value 1 as it signifies the control group. Data were analyzed using a non-parametric Kruskal–Wallis test with Dunn’s post hoc test. Median with interquartile range is shown. Asterisks mark significant differences * *p* < 0.05.

In order to validate the microarray results, we performed real-time qPCR reactions for randomly selected genes *FOXP3*, *MAPK3*, and *LEF1* (Figure 7). The obtained validation results showed the differences in the gene expression between CVID and control groups as well as between SIgAD and control groups, and confirmed the microarray results. Among the selected genes, only *LEF1* was differentially expressed when CVID and SIgAD patients were compared with healthy controls (fold change –3.91 and –2.43, respectively).

**Figure 8:**
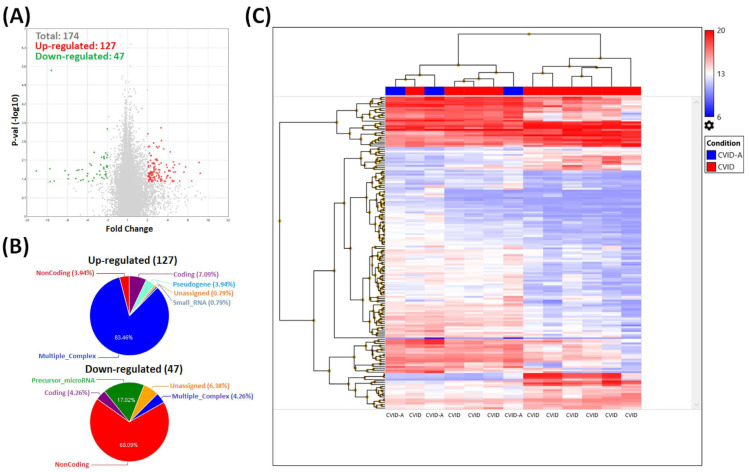
Results of microarray analysis of T_reg_ cell transcriptome profiles in CVID with (CVID-A) and without (CVID) autoimmune symptoms. (**A**) Volcano plot showing all 174 differentially expressed transcripts (*p*-values < 0.05). Red spots represent up-regulated and the green ones represent down-regulated genes. The grey colored dots represent the non-significantly differentially expressed genes. (**B**) The pie graphs showing the percentage of up-regulated (**top**) and down-regulated (**bottom**) genes, belonging to several categories. (**C**) Hierarchical clustering analysis of the CVID patient samples of all 174 differentially expressed genes (*p* value < 0.05, fold change > 2 or <−2). Each row represents one of the 174 genes and each column is a separate patient’s sample. A colored representation of the relative intensity is shown such that a red color indicates high and blue color indicates low expression values.

Additionally, due to the occurrence of autoimmune diseases in several CVID and SIgAD patients, we performed the analysis of T_reg_ gene profiles separately in the groups of CVID and SIgAD patients, taking into account the coexistence of accompanying autoimmunizations (Table 1). As a result, in the CVID group, a total of 174 genes were differentially expressed when compared to patients with (CVID-A) and without (CVID) additional diseases. Among them, 127 were up- and 47 were down-regulated (Figure 8A). The majority of up-regulated genes belonged to multiple complex groups (83.46%), while the rest were coding (7.09%), noncoding (3.94%), pseudogenes (3.94%), and small RNAs (0.79%). Among the down-regulated genes, 68.09% belonged to the noncoding group, 17.02% were microRNA precursors, 4.26% were coding, and 4.26% belonged to the multiple complex group, while the rest were unassigned (6.38%) (Figure 8B). Figure 8C shows the heat map of all 174 mentioned transcripts selected when *p*-value < 0.05 and fold change ± 2. For further analysis we selected two genes reported as important for T_reg_ functions: *IRF1* and *STAT1*. Their characteristics are presented in Table 3.

**Figure 9:**
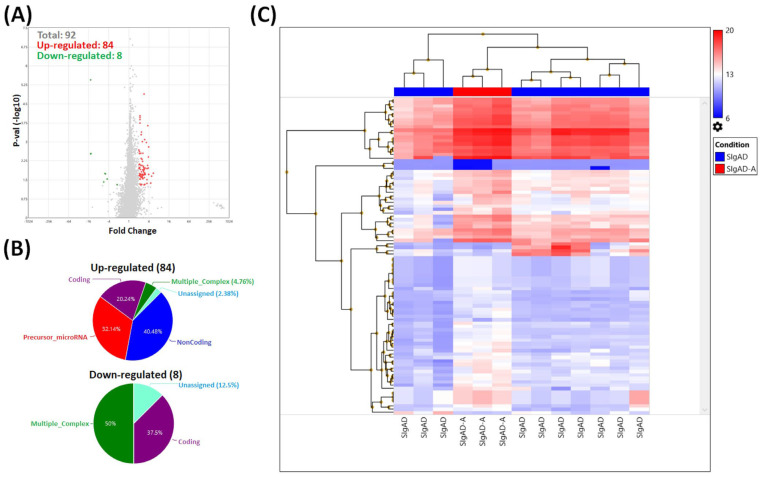
Results of microarray analysis of T_reg_ cells’ transcriptome profiles in SIgAD with (SIgAD-A) and without (SIgAD-NA) autoimmune diseases. (**A**) Volcano plot showing all 92 differentially expressed transcripts (*p*-values < 0.05), with red spots representing the up-regulated and the green ones representing the down-regulated genes. The grey colored region represents the non-significantly differentially expressed genes. (**B**) The pie graphs showing the percentage of up-regulated (**top**) and down-regulated (**bottom**) genes belonging to one of several categories. (**C**) Hierarchical clustering analysis of the SIgAD patients’ samples of all 92 differentially expressed genes (*p* value < 0.05, fold change > 2 or <−2). Each row represents one of the 92 genes, and each column is a separate sample. A colored representation of the relative intensity is shown such that a red color indicates high and blue color indicates low expression values.

In SIgAD sub-groups, a total of 92 genes were differentially expressed when patients with (SIgAD-A) and without (SIgAD) autoimmunization were compared. Among them, 84 were up- and 8 were down-regulated (Figure 9A). Regarding up-regulated transcripts, the percentage distribution of the individual groups was as follows: 40.48% belonged to the noncoding group, 32.14% to microRNA precursors, 20.24% were coding, 4.76% were in multiple complex groups, and 2.38% were unassigned. Among down-regulated genes, half comprised the multiple complex category, while the rest were coding (37.5%) and unassigned (12.5%) (Figure 9B). Figure 9C shows the heat map of all 92 mentioned transcripts selected when *p*-value < 0.05 and fold change ± 2. For further analysis we selected known transcripts belonging to the coding and multiple complex groups, with fold change > 3 or <−3. As a result, four genes were selected: *IFIT1*, *MX1*, *IFI6*, and *IFI44L*. Their characteristics are presented in Table 4.

## 4. Discussion

The role of T_regs_ in the development and progression of CVID has been previously considered. Several researchers, starting with Fevang et al., demonstrated a lower frequency of T_reg_ cells in patients with CVID; however, opposite results have also been published [17,18,19,20,21,22]. It has been speculated that the discrepancy observed in the number of T_regs_ in patients with CVID may be due to coexisting autoimmune diseases in some patients. Indeed, several previous studies have confirmed that in CVID patients with symptoms of autoimmunization, a significant decrease in Foxp3 mRNA expression and the proportion of T_reg_ cells in comparison to controls was observed [23,24,25,26,27]. In our study, a significantly lower level of circulating T_reg_ lymphocytes was observed in all patients with CVID compared to healthy controls (Figure 2A). However, this observation did not seem to be related to concomitant autoimmune diseases (Figure 2B). Nonetheless, the lack of statistically significant differences may result from the small number of patients in each subgroup (3 patients with and 10 without autoimmunization). Previous studies suggested that T_regs_ are major helpers for the induction and maintenance of B cells, eliciting a T cell-dependent IgA response in the intestinal mucosa, but no indirect association between T_regs_ and SIgAD has been described. This seems to be confirmed by our study, as in patients with SIgAD, the mean level of circulating T_regs_ was similar to that observed in age-matched healthy control subjects (Figure 2A). However, the latest meta-analysis of GWAS-based studies performed by Bronson et al. revealed that one of the pathways that may lead to IgA deficiency was connected to T_reg_-associated genes [61].

To the best of our knowledge, there is no data about gene expression profiles of T_reg_ cells isolated from children with SIgAD or CVID. Here, we have shown for the first time that gene expression patterns of T_regs_ isolated from patients with these two immunodeficiencies differ from those isolated from healthy subjects. Interestingly, in the CVID group, the majority of DEGs were down-regulated, while in SIgAD, they were up-regulated when compared to the same control group. T_regs_ isolated from CVID patients, when compared to T_regs_ from healthy controls, were enriched in Th response-associated genes, including T cell receptor signaling pathways, and associated with Th17 cell differentiation. T_reg_ cells were shown to regulate all types of Th response, including Th1, Th2, and Th17, and the mutual association of Th and T_reg_ cells currently seems far more complex than the primary concept of effector Th cells and T_reg_ cells inhibiting each other [62,63]. It is thus possible that impaired Ig production in CVID patients is associated with dysregulated Th response control by their T_reg_ cells. Alternatively, Th response-related gene enrichment in T_reg_ cells might constitute a counterbalance mechanism, trying to stimulate aberrant Ab production. 

Pathway enrichment analysis of T_regs_ isolated from SIgAD patients detected DEGs associated with the innate immune response, including innate receptor signaling pathways, e.g., NOD-like, C-type lectin like, or Toll-like receptors, as well as TNF signaling pathways or apoptosis, when compared to healthy controls. NOD-like, C-type lectin like, and Toll-like receptors are pattern recognition receptors (PRRs), playing crucial roles in recognition of pathogens and induction of the immune response [64]. These observations might be associated with higher viral infection rates or its more severe course, as was observed during the COVID-19 pandemic [65]. This phenomenon might be associated with the exposition of SIgAD patients to higher viral loads, due to the lack of protective IgA levels in the upper respiratory tract, resulting in heavy inoculation [65].

Recent data suggest that CVID and SIgAD patients may have similar or identical genetic backgrounds [1]. This claim seems to be supported by the observation that both CVID and SIgAD patients share clinical manifestations; both disorders have been observed in the members of one family, and one can progress into the other [66,67,68]. Therefore, in our study, we also focused on DEGs that were similarly regulated in both studied types of immunodeficiencies when compared to the same control group. Thus, we found that five genes were differentially expressed both in CVID and SIgAD, presenting the same regulation pattern: *CAMK4* and *IL6ST* were down-regulated, while *OGFRL1*, *ATP6V1B2*, and *TBNAIP2* were up-regulated. The gp130 receptor encoded by *IL6ST* forms a receptor complex with several cytokines, including IL-6, IL-27, and IL-11 [69]. A previous study showed that high expression of *IL6ST* identifies a specific T_reg_ subset with reduced suppressive capacity ex vivo, and a subsequent blockade of gp130 was able to restore it to normal levels [70]. Here, we observed lower *IL6ST* expression levels in PID patients than in healthy children, which may suggest increased T_reg_ suppressive function. However, additional functional studies are required to confirm this observation. Another gene up-regulated in both CVID and SIgAD patients compared to controls was calcium/calmodulin-dependent kinase IV (*CAMK4*). CAMK4 is a serine/threonine kinase regulated by intracellular calcium levels, which is important for activating transcription factors downstream of T cell receptor signaling [71]. Many previous studies have suggested that CAMK4 is a central molecule that contributes to multiple pathological pathways in T cells in patients with systemic lupus erythromatosus (SLE) [72]. It was shown that SLE T cells are characterized by increased CAMK4 activity, while Camk4 global knockdown improves autoimmunity in mice [73]. Camk4 inhibition enhanced mouse T_reg_ cell differentiation and function in vitro, impaired T helper 17 (TH17) cell differentiation, and increased IL-2 production by conventional T cells [74]. Moreover, CAMK4 advances aerobic glycolysis and promotes TH17 cell differentiation by controlling the activity of pyruvate kinase M2 [75]. Nevertheless, the exact mechanism by which CAMK4 negatively affects T_reg_ cell function remains unknown. In our study, we observed decreased expression levels of CAMK4 in PID patients, which may reflect an attempt to compensate for the abnormal functioning of T_regs_. In the cases of *OGFRL1*, *ATP6V1B2*, and *TBNAIP2*, there is no data regarding their connection with CVID, SIgAD, or T_reg_ cells. 

Additionally, we have evaluated the role of T_reg_ cells in autoimmune diseases among SIgAD and CVID patients by separately comparing the gene expression profiles of T_regs_ isolated from patients with and without autoimmunity for both types of analyzed immunodeficiencies. As a result, no common genes for both analyzed groups of PID with autoimmunization were identified. In SIgAD patients with concomitant autoimmune diseases, decreased expression levels of genes related to the type I interferon (IFN) pathway, including *IFIT1*, *MX1*, *IFI6*, and *IFI44L*, were observed. It was previously shown that dysregulation of IFN-stimulated gene expression can cause dysfunctional antiviral responses and autoimmune disorders [76]. Indeed, the genes reported in our study have previously been associated with various autoimmune diseases (Table 4). Moreover, according to our observations, SIgAD patients with autoimmunization are usually less prone to viral infections, which may result in a weaker stimulation of the IFN pathway and lower IFN levels in these patients. On the other hand, it was shown that type I IFN signaling can exert beneficial effects by acting on T_regs_ to downmodulate their suppressive functions, resulting in a more effective antiviral response and impaired antitumor immunity [77]. In our study, a connection with the IFN pathway was also observed in CVID patients with autoimmunizations. When compared to CVID patients without autoimmunizations, up-regulation of *STAT1* and *IRF1* gene expression was observed. The main role of STAT1 is to transmit IFN signals, activating the antiviral immune response. It also regulates Th1 cytokine production, proliferation, and apoptosis of immune cells [78]. Elevated expression of STAT1 mRNA was reported in lupus nephritis and correlated with disease progression [79]. *IRF1* gene expression was also indicated to associate with autoimmune disease risk [80]. 

There are some limitations of our study. Firstly, it might be claimed that microarray analysis was performed in low-number groups of patients. The number of children with CVID is limited by the low prevalence of this type of immunodeficiency, while most patients with SIgAD are asymptomatic; thus, they do not attend our Outpatient Clinical Immunology Unit. Autoimmunity occurrence within CVID and SIgAD patients corresponded to the literature data [2,81]. The accompanying autoimmune diseases in the patient cohorts are very variable [2,81], thus the possible association of the aberrant IFN signaling pathway with autoimmunity requires further studies on larger groups of patients that are more concise in concomitant autoimmune diseases. Secondly, in our study the F:M ratio of the CVID group (5:8) does not match the SIgAD and control groups. Nonetheless, it resembles a bimodal sex distribution in CVID which was found by Janssen et al., with male predominance in children with CVID (62%) and female predominance in adults (58%) [82]. Finally, the microarray analysis results would be greatly supplemented by T_reg_ functional analysis. However, the limited amount of biological material obtained in this study was insufficient for any additional analysis. 

## 5. Conclusions

Our findings suggest that the gene signature of T_reg_ cells isolated from SIgAD and CVID patients differ from age-matched healthy controls and from each other, presenting transcriptional profiles enriched in innate immune or Th response, respectively. The occurrence of autoimmunity in both PID types seems to be associated with class I IFN signaling pathways in T_reg_ cells. 

## Figures and Tables

**Table 1:** Characteristics of patients and control group.

	CVID	SIgAD	Control Group
Number of children	13	13	11
Age (years ± SD)	10.9 ± 3.4	8.36 ± 3.1	6.25 ± 4.35
Sex (F/M)	5/8	7/6	6/5
Familial history of immunodeficiencies	1/13	2/13	0/11
Accompanying diseases	2 patients with thrombocytopenia, 1 patient with ulcerative colitis	1 patient with celiac disease, 1 patient with juvenile arthritis, 1 patient with Sjögren disease	0

**Table 2:** Characteristics of genes differentially expressed both in CVID and SIgAD patients when compared to healthy controls. Functional annotations were obtained from the UniProt database [30].

Gene Symbol (Gene Name)	Fold Change in CVID When Compared to Control (*p* Value)	Fold Change in SIgAD When Compared to Control (*p* Value)	Functional Annotation
*CAMK4* (calcium/calmodulin-dependent protein kinase IV)	−2.65 (0.0043)	−2.31 (0.0253)	Calcium/calmodulin-dependent protein kinase involved in the calcium-triggered CaMKK-CaMK4 signaling cascade, regulating, mainly by phosphorylation, the activity of several transcription activators, such as CREB1, MEF2D, JUN, and RORA, which play a pivotal role in immune response, inflammation, and memory consolidation.
*IL6ST* (interleukin 6 signal transducer)	−2.35 (0.0018)	−2.09 (0.0466)	Gp130 is involved in the receptor complex formation in the case of several cytokines, including IL-6, IL-27, and IL-11. Gp130 functional impairment in hematopoietic cells results in defective lymphocyte development.
*OGFRL1* (opioid growth factor receptor-like 1)	5.18 (0.0438)	5.62 (0.0038)	No UniProt annotations available.
*ATP6V1B2* (ATPase, H+ transporting, lysosomal 56/58 kDa, V1 subunit B2)	2.33 (0.0454)	2.71 (0.0184)	Non-catalytic subunit of the V1 complex of vacuolar(H+)-ATPase (V-ATPase). V-ATPase is associated with acidifying and maintaining the pH of intracellular compartments, and in some cell types is responsible for acidifying the extracellular environment.
*TNFAIP2* (tumor necrosis factor, alpha-induced protein 2)	3.16 (0.0354)	2.03 (0.0481)	May play a role as a mediator of inflammation and angiogenesis.

**Table 3:** Characteristics of top selected genes related to T_reg_ cells when CVID patients with accompanying diseases were compared with patients without additional diseases.

Gene Symbol	Group	Fold Change (*p* Value)	Description	Selected Association with Autoimmune Diseases
*IRF1*	Multiple Complex	4.63 (0.0455)	interferon regulatory factor 1	pemphigus [31], systemic sclerosis [32], rheumatoid arthritis [33], localized scleroderma [34]
*STAT1*	Multiple Complex	3.61 (0.0219)	signal transducer and activator of transcription 1	systemic lupus erythematosus [35], Sjögren’s syndrome [36], rheumatoid arthritis [37]

**Table 4:** Characteristics of top selected genes when SIgAD patients with autoimmune diseases were compared with patients without autoimmunizations.

Gene Symbol	Fold Change (*p* Value)	Description	Selected Association with Autoimmune Disease
*IFIT1*	−5.68 (0.0383)	interferon-induced protein with tetratricopeptide repeats 1	systemic lupus erythematosus [38], primary Sjögren’s syndrome [39], autoimmune demyelinating CNS disorders [40], ankylosing spondylitis [41], Anti-neutrophil cytoplasmic antibody (ANCA)-Associated Vasculitis (AAV) [42], juvenile dermatomyositis (JDM) [43], systemic sclerosis [44], experimental autoimmune encephalomyelitis [45], type 1 diabetes [46]
*MX1*	−5.23 (0.0176)	MX dynamin-like GTPase 1	dermatomyositis [47], systemic lupus erythematosus [48], Sjögren syndrome [49], juvenile idiopathic arthritis [50], type 1 diabetes [51], mixed connective tissue disease [52], primary antiphospholipid syndrome [53]
*IFI6*	−5.06 (0.0184)	interferon, alpha-inducible protein 6	dermatomyositis [54], systemic lupus erythematosus [55], Hashimoto’s thyroiditis [56], rheumatoid arthritis [57]
*IFI44L*	−4.42 (0.0294)	interferon-induced protein 44-like	primary Sjogren’s syndrome [39], systemic lupus erythematosus [58], rheumatoid arthritis [59], (ANCA)-Associated Vasculitis (AAV) [42], mixed connective tissue disease [52], systemic sclerosis [60]

## Data Availability

The microarray data will be submitted to the GEO database.

## Supplementary Materials

The following supporting information can be downloaded at: https://www.mdpi.com/article/10.3390/cells13050417/s1. Figure S1: Determination of the absolute number of Treg cells. First, the whole blood samples were stained in TruCOUNT™ Tubes (BD Biosciences, CA) with anti-CD3-FITC and anti-CD4-PE (BD Biosciences) mAbs and the CD3/CD4-positive cells were gated (10.000 cells in each sample) (plot A). T_reg_ cells gating strategy among PBMC population was as follows: based on granularity (SSC pa-rameter) and high CD4 expression, the CD^4+^ cell population was determined (plot B1). Of these, cells with expression of CD25 antigen were gated (plot B2). In the upper square on plot B3, CD^4+^CD^25+^Foxp3+ population was considered T_reg_ cells. The example staining of SIgAD patient is shown. Table S1: The raw data of gene expression profiling of T_reg_ cells isolated from children with SIgAD, CVID and healthy controls. In all analyzed comparisons, DEGs were identified when 2-fold change and *p*-value < 0.05 served as cut-off criteria.
