# Supplementary figures and images for: Gene Signature of Regulatory T Cells Isolated from Children with Selective IgA Deficiency and Common Variable Immunodeficiency
Source: Cells. 2024 Feb 27;13(5):417. doi: 10.3390/cells13050417 (PMC10930802; doi:10.3390/cells13050417)

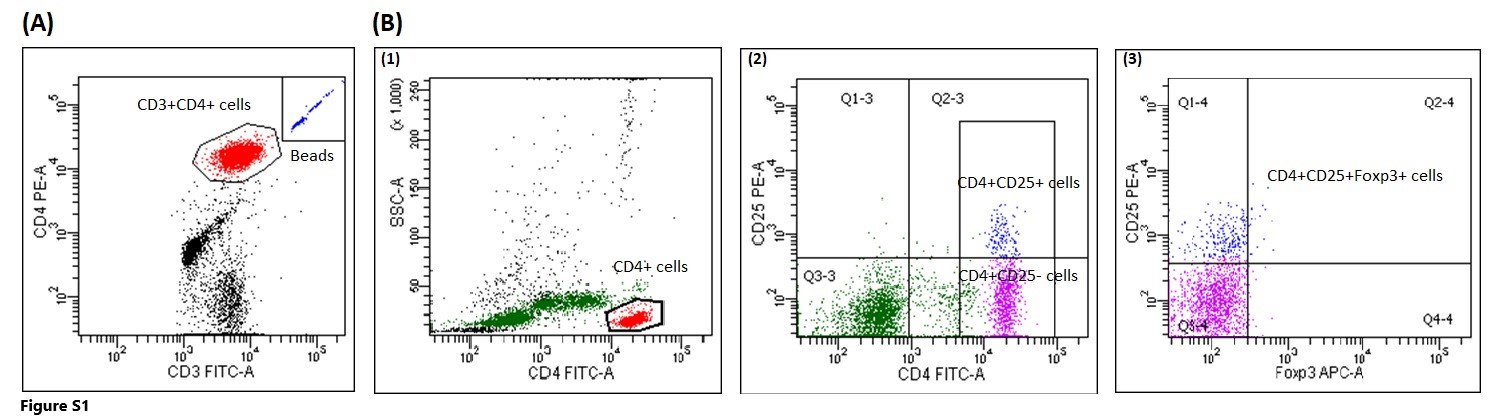

Supplement: Supplementary file 1 [file cells-13-00417-s001.zip › Supplementary Figure S1.jpg]
